# A simulation-based role-reversal training program improves knowledge, skills, and job satisfaction of endovascular scrub nurses

**DOI:** 10.1016/j.sipas.2026.100335

**Published:** 2026-02-19

**Authors:** Jonathan Lawaetz, Maj Siercke, Rebecca Skov, Lars Konge, Isabelle Van Herzeele, Jonas Peter Eiberg

**Affiliations:** a Department of Vascular Surgery, University Hospital Copenhagen - Rigshospitalet, Copenhagen, Denmark; b Copenhagen Academy for Medical Education and Simulation (CAMES), Centre for Human Resources and Education, Copenhagen, Denmark; c Department of Clinical Medicine, University of Copenhagen, Denmark; d Department of Thoracic and Vascular Surgery, Ghent University Hospital, Ghent, Belgium; e SPL Publishing, Journal of Nursing Research & Professional Knowledge. Denmark

**Keywords:** Role-reversal training, Simulation-based education, Endovascular scrub nurses, Technical skills, Non-technical skills, Job satisfaction

## Abstract

**Objectives:**

This study assessed the effectiveness of a modified simulation-based education (SBE) program, originally designed for vascular surgical trainees, adapted for endovascular scrub nurses (ESNs). The goal was to enhance their technical and non-technical skills and explore the program’s impact on operating room (OR) teamwork and workflow.

**Materials and methods:**

A mixed-methods design was used. Eleven experienced ESNs—each with experience from approximately 100 endovascular procedures in the past 2–3 years—participated in role-reversal simulations, practicing peripheral endovascular procedures in the role of the surgeon under the guidance of a supervisor. Quantitative assessments included the Global Rating Scale (GRS) and an Examiner’s Checklist for technical skills, while semi-structured interviews provided qualitative insights into non-technical domains such as teamwork, workflow, and job satisfaction.

**Results:**

The program significantly improved ESNs' technical skills, with GRS scores increasing from median [IQR] 19.0 [14.0; 24.0] to 43.0 [32.0; 44.0] *(p=.*004) and Examiner’s Checklist scores rising from 49.0 [45.0; 58.0] to 73.0 [65.0; 78.0] *(p=.*004). Qualitative findings, derived from the themes of learning potential, job satisfaction, culture, feedback, overwhelming, and COVID-19, further demonstrated improvements in non-technical domains, including understanding of procedural sequences, teamwork, workflow, and job satisfaction. Participants reported better anticipation of surgeons' needs and a more collaborative atmosphere in the operating room.

**Conclusions:**

The role-reversal simulation-based education program for ESNs improved technical skills and strengthened teamwork and collaboration in the operating room. These findings highlight the potential of role-reversal training to enhance surgical performance and job satisfaction among OR staff.

## Highlights

• Role-reversal simulation-based education program enhances endovascular scrub nurses' technical skills.
• Rating Scale scores increased from 19.0 to 43.0 (max 55), Checklist scores from 49.0 to 73.0 (max 85).
• Improved understanding of procedures boosts teamwork and job satisfaction.
• Endovascular scrub nurses reported better anticipation of surgeons' needs, fostering collaboration.
• Potential for role-reversal training to improve surgical outcomes and job satisfaction.

## Introduction

Growing demands for patient safety and outcomes require highly skilled surgical teams, where scrub nurses play a central role in ensuring efficiency and safety [[1], [2], [3], [4], [5]] Well-tuned teamwork in the OR is essential for patient safety and rational use of resources; the growing complexity of surgical procedures and increased implementation of new technologies further compound this [3] Endovascular scrub nurses (ESNs) must understand both the technical workflow and procedural sequence [6,7] Simulation-based education (SBE) effectively improves endovascular competence among vascular trainees and is increasingly integrated into training curricula [[8], [9], [10], [11], [12], [13], [14]] SBE combines procedural training and teamwork in a safe environment, improving both technical and interpersonal skills [[15], [16], [17]] Previous studies show that role-reversal training, where ESNs complete an endovascular aortic repair (EVAR) program originally developed for trainees, reduces errors and stress [18] However, its broader applicability and how ESNs perceive this approach remain unclear [[19], [20], [21]] This study aims to assess the effectiveness of an adapted interprofessional endovascular skills training program for experienced ESNs in terms of improvement in technical and non-technical abilities, and it seeks to understand the derivative effects of such a program on daily activities and teamwork in the actual OR.

## Materials and methods

### Study design

This prospective single-centre mixed-methods study evaluated a simulation-based role-reversal training program for endovascular scrub nurses (ESNs) between March 2020 and March 2021. Ethical approval was waived by the Capital Region of Denmark’s Ethics Committee (Ref. H-21,063,651). All participants provided written informed consent. The study was conducted in accordance with the World Medical Association Declaration of Helsinki. Data handling complied with GDPR regulations.

### Participants

The study included experienced ESNs, all employed at the same Department of Vascular Surgery with approximately 450 endovascular procedures for lower-limb ischemia annually. Each ESN had assisted approximately 100 PAD procedures within the last 2–3 years. Only experienced ESNs were approached and invited face-to-face and by email. Participation was voluntary, and ESNs were enrolled upon consent. None of the invited ESNs declined the invitation. This was done to ensure a uniform baseline of procedural familiarity and to allow the training to focus on role-reversal understanding and interdisciplinary teamwork rather than basic scrub-nurse competencies. This approach also ensured a homogeneous study group, thereby minimising potential confounders related to differences in experience. Vascular surgical consultants performed all procedures in the real OR.

### Intervention

The SBE program in basic endovascular skills was initially designed for VSTs at the onset of their endovascular career. The program is derived from the 'PROficiency-based Stepwise Endovascular Curricular Training program' (PROSPECT)[22] and consists of four modules incorporating e-learning and simulation cases. The modules covered 1) basic endovascular skills, 2) iliac artery disease, 3) superficial femoral artery disease, and 4) postoperative management. The modules included five simulation cases, each completed four times. Three hours were allocated to each hands-on simulation module. Simulations were conducted on the Angio Flex II system (SurgicalScience, Sweden).

The instructor-to-trainee ratio was 1:1. The instructor, a physician certified PROSPECT trainer with > 500 simulation cases, controlled the C-arm upon request. ESNs independently planned the procedure and device selection. Instructors provided concurrent and terminal feedback to support real-time learning and post-session reflection [23] Pre- and post-course tests were observational only.

### Outcome measures

#### Quantitative assessments

Knowledge assessment was done with standardised multiple-choice questionnaires (MCQ) pre- and post-course and at the end of each module. As in the original PROSPECT programme, passing each module’s MCQ was required before progression [22] In contrast, benchmark performance in simulation cases was not mandatory. Technical skills were evaluated pre- and post-course with a standardised simulation case on a common iliac artery lesion. They were assessed by the same instructor using a Global Rating Scale (GRS) and an Examiner’s Checklist, both rated 1–5 with a maximum of 55 and 85 points, respectively.

#### Qualitative assessments

Two semi-structured focus-group interviews (*n* = 5 and 6) were conducted 1–2 months after course completion in a confidential setting to explore participants’ experiences with the training and its impact on their daily practice. Interviews were led by an independent and experienced interviewer (MS), a registered nurse from the same department who was familiar with the ESNs but not involved in the SBE program, using pre-defined topics (Table 1), with assistance from a trained observer ensuring consistency. Sessions were audio-recorded, transcribed verbatim, and analysed inductively in NVivo (QSR International, LLC, USA) by JL, RS, and MS following the approach of Elo et al. [24] and applying Malterud’s concept of “information power” to determine sample adequacy [25] Accordingly, sample adequacy was guided by information power rather than formal assessment of data saturation.

**Table 1:** Topics of semi-structured interviews conducted with endovascular scrub nurses after receiving role-reversal simulation training in basic endovascular skills.

• Course relevance and effectiveness for daily practice in the operating room • Understanding and clarity of the course objectives • Experience of the course structure and delivery • Impact of the course on job engagement and satisfaction • Changes in workflow and team dynamics within the angio-suite post-training • Challenges in applying new skills in practice • Observations of improved procedural flow and reduced stress • Experiences of reduced errors and their reflections on teamwork and communication.

The table summarises the pre-set topics explored during the semi-structured interviews conducted with endovascular scrub nurses after completing the role-reversal simulation training.

### Statistics

All analyses were performed in R (version 4.4.0; R Foundation for Statistical Computing, Vienna, Austria) using RStudio (version 2024.12.0 + 467; Posit Software, PBC). As the Global Rating Scale (GRS) and Examiner’s Checklist are ordinal and the sample size was limited, normality was not assumed, and data were summarised as median [IQR]. Pre- and post-course comparisons were conducted using the Wilcoxon signed-rank test for paired data. All tests were two-sided, and p < 0.05 was considered statistically significant.

### Reporting guidelines

The study follows the STROBE and COREQ reporting guidelines for quantitative and qualitative components, respectively.

## Results

This study included 11 ESNs. The results are divided into quantitative and qualitative data. The quantitative data outline progression in pre- and post-SBE knowledge and technical skills, and the qualitative data should improve understanding of the derivative effects of SBE on daily activities, including real procedures in the OR.

### Quantitative data

The pre- and post-MCQ scores significantly increased from median [IQR] 12.0 [11.0; 13.0] to 14.0 [13.0; 16.0] (*p* = .012). Significant improvement was also observed in technical skills, as evidenced by the GRS with an increase from median [IQR] 19.0 [14.0; 24.0] to 43.0 [32.0; 44.0] (*p=*.004), the Examiner's checklist with an increase from median [IQR] 49.0 [45.0; 58.0] to 73.0 [65.0; 78.0] (*p=*.004), and reduction in the Procedure and Fluoroscopy times, from median [IQR] 39.2 [28.0; 42.5] to 18.4 [16.6; 21.5] minutes, and 21.0 [14.2; 27.5] to 10.1 [9.1; 13.2] minutes, respectively (*p=*.004 and *p*=.005) (Table 2).

**Table 2:** Comparison of pre and post simulation based educational-course in role-reversal basic endovascular skills for endovascular scrub nurses, showing simulator metrics, technical performance, and multiple-choice questionnaire.

	Pre Median *(IQR)*	Post Median *(IQR)*	*Wilcoxon p-value*
Procedure time (mins)	39.2 [28.0; 42.5]	18.4 [16.6; 21.5]	.004
Fluoroscopy time (mins)	21.0 [14.2; 27.5]	10.1 [9.1; 13.2]	.005
Contrast volume (mLs)	69.0 [40.0; 88.0]	55.0 [45.0; 80.0]	.838
Number of Roadmaps	8.0 [3.0; 12.0]	7.0 [5.0; 8.0]	.798
Global Rating Scale (max 55)	19.0 [14.0; 24.0]	43.0 [32.0; 44.0]	.004
Examiner’s Checklist (max 85)	49.0 [45.0; 58.0]	73.0 [65.0; 78.0]	.004
Multiple-choice Questionnaire (max 20)	12.0 [11.0; 13.0]	14.0 [13.0; 16.0]	0.012

IQR = interquartile range. *m* = Minutes. mL =millilitres. Significant p-values < 0.05.

### Qualitative data

Six themes emerged from the inductive qualitative analysis: 1) Learning potential, 2) Job Satisfaction, 3) Culture, 4) Feedback, 5) Overwhelming, and 6) COVID-19.

#### Learning potential

The ESNs reported improvements in their understanding of procedures and tasks.

One ESN noted the course's timing as optimal given the experience level, "*I would not have thought it made sense to take the course before I started working in the OR”* (F). The crucial role of combining theory with practical simulations was highlighted, "*If I had only received the theory-, without the following hands-on sessions (ed: SBE), then I don't think I would have remembered as much – it simply wouldn't have stuck"* (I). Participants felt a sense of progress and personal growth, "*I really felt that I was improving over those four simulations" (A),* and they noted an increased ability to anticipate procedural steps in the real OR, *"Okay, we are this far in the process, the next step would be…*" (I). The experience was deemed essential for ESNs, with one suggesting, "*I think it should be mandatory for new people that they go through something like this*" (H). The desire for more SBE was consistent, "*Yes, I really think it has been interesting and fun. I wish we could spend more time doing simulations…*" (D). These statements collectively suggested that SBE improved the ESNs' technical skills and contributed to a more collaborative and efficient atmosphere in the OR.

#### Job satisfaction

The ESNs experienced enhanced job satisfaction, marked by a notable sense of achievement and engagement. "*It was like 'Yes, I can figure it out,' right…Getting that sense of success, I think, was very important for my engagement*" (A). The positive atmosphere in the OR during regular workdays was crucial for accomplishment and satisfaction, "*Once we got started, I found it fun, and we felt like 'Ah, I made it.' There was great support from the others…*" (J). Improved understanding of the work tasks benefits job satisfaction and collaboration with the entire team. As one ESN noted, "*Yes, I feel it's much more enjoyable to be down in the OR now…I much better understand the reason for why the surgeons need the different tools now…*" (J) and "*Now it's like…you know what's next. It's much more fun to be a part of it down there* (in the OR)*"* (J). Another pointed out an enhanced ability to ask precise, relevant questions after the SBE, "*It creates a better atmosphere at the table when the doctors can sense 'Okay, that question is well-thought-out.'*" (A). The ESNs asked for more hands-on simulation to improve their understanding of increasingly complex endovascular procedures.

#### Culture

Initially, ESNs doubted that training in surgeon-specific tasks would benefit them in their specific tasks. As stated, "*It was a bit far-fetched to think that we would become better assistants by being able to perform the task*" (D). Some ESNs questioned the doctor-centric training material, "*I think many of the questions were doctor-based. That is, they are not nursing-based*" (C). Some further highlighted a cultural difference between doctors' and nurses' professional competence, “*.. these classifications, they were extremely difficult to remember…We really haven't dealt with them ever… It might well be that doctors have the consideration in their head, but we don't see it."* (J). These statements underscored the importance of more inclusive and cognizant training, including both the nurses' and doctors' roles, and maybe a better introduction to the ideas behind “role reversal training”.

#### Feedback

The ESNs appreciated concurrent supportive and constructive feedback, "*I felt more secure. It was more supportive guidance, which I really appreciated*" (B). Comparing the first session (pre-test without feedback to the later sessions (with feedback), the ESN felt more coached, "*It was quite clear from the first session* (pre-test without feedback) *where the instructor was completely silent, to feeling not just guided but properly coached*" (C). The concurrent constructive feedback heightened motivation, "*When the instructor began to provide constructive feedback, it really motivated me*" (A). Personalised feedback was highly valued, "*Yes, I think the instructor was very skilled at giving feedback…*" and "*The way the instructor gave tips…made us more confident in the situation*" (D). The ESN valued reflective discussions, and clarifying test questions were helpful, "*Regarding the multiple-choice tests…he would explain pedagogically…the instructor was just great at making me feel not so clueless."* (B).

#### Overwhelming

Some ESNs perceived SBE participation as enforced and overwhelming, "*It wasn't a course where you felt you had a choice to attend or not*" (D). The ESN experienced challenges understanding the course language, "*I found it really hard to understand this professional English"* (A). In addition, the e-learning material was considered complex "*Yes, I thought it was complex*" (J), particularly for the less experienced ESNs. "*..I am the one with the least experience here, so for me, it was a huge task*" (B). The unprepared pre-test without feedback was poorly received, "… *that pre-test simulation… It was actually an unpleasant experience*" (B), and apparently its purpose was unclear, "..*I recommend that they made more of an effort to say 'This is just to see the starting point'*" (J) and “..*I somehow feel I'm being judged on something that I don't really have a basis for knowing 100 %*”. Balancing theoretical learning with clinical duties was a struggle, "*It's been a bit hard sometimes to manage both the theoretical part and taking care of clinical tasks*" (F).

#### Covid-19

The impact of the COVID-19 pandemic affected the ESNs' perception of the course. There was tension between professional obligations and the pandemic's overwhelming impact, making SBE priorities misaligned. One ESN expressed, “*During this COVID time…I've found it incredibly difficult…I've also felt a bit irritated about it…because I've thought, 'Is this what's important right now?”* (K).

## Discussion

The ESNs demonstrated significant improvement in knowledge and technical skills following the SBE. This was evident in key areas such as procedure and fluoroscopy times, GRS, Examiner's Checklist and MCQs. Two novel aspects of this study were the clinical applicability of the principle of role reversal training and the fact that the ESNs' improvement was comparable to that of VSTs, the SBE program's original target. As recently shown by Soenens et al.[26], VSTs improve significantly after receiving basic endovascular SBE (PROSPECT) comparable to that offered in the present study. The ESNs in the present study improved similarly on the majority of the metrics in the amended SBE program despite not being required to reach a predefined benchmark.

Beyond the improvements in knowledge and technical skills, the ESNs also reported better non-technical abilities, such as anticipation, communication, and teamwork. In our study, these enhancements appeared to stem from a deeper technical understanding, reflecting the interdependence between technical and non-technical competence. Similar effects have been observed by Gowda et al. ^20^, where nurses receiving role-reversal SBE in microsurgery improved their procedural understanding and daily performance, and by Satkunanatham and Sechachalam, who found enhanced anticipation of surgeons’ needs [27] This phenomenon—technical training improving non-technical performance—has been further supported by Brunckhorst et al. and Hull et al., highlighting a strong correlation between technical proficiency and teamwork [16,17]

Differences between ESNs’ and doctors’ educational backgrounds posed challenges, as evaluations and terminology were not always aligned with nursing practice. This reflects cultural differences in training approaches and underscores the need to adapt surgeon-focused SBE to nursing contexts to ensure relevance and applicability.

In the SBE program, varied feedback methodologies caused distinct responses from the nursing participants, diverging from prior observations gathered when using the same SBE program with vascular surgical trainees. This divergence in feedback likely mirrors the disparate training conventions inherent to these two professional cohorts. Initially, particularly during the pre-and post-test, the ESNs felt uncomfortable while being observed and assessed without receiving feedback. This “unsafe” atmosphere was not conducive to the learning environment, underlined by the participants' very positive statements regarding the more dialogic-based and supportive concurrent feedback used later in the program. Initially, the training strictly followed the basic endovascular skills program designed for VST, causing unease among ESNs with its feedback style and assessment. Adapting the training to include concurrent feedback and altering the passing requirements proved beneficial for the overall reception of the program. This change aligns with Schober et al.'s, showing equal effects of concurrent and terminal feedback, and contrasts with Von Heukm et al.'s findings, in which concurrent feedback yielded worse results than terminal in resuscitation training [28,29] This suggests that feedback methods may need to be tailored to the training context and the learners' backgrounds.

The ESNs experienced a sense of obligation to participate in training without fully understanding its purpose. The complexity of the e-learning exacerbated by the language barrier, further contributed to them feeling overwhelmed. This highlights the need to better communicate and share the objectives of this SBE program, its content and possible didactic considerations. This is paramount to creating a psychologically safe environment facilitating SBE, which is crucial for effective learning. Psychological safety allows participants to engage without fear of embarrassment or retribution [30] Failing to provide psychological safety may lead to feelings of incompetence, frustration and psychological unsafety, which can be detrimental to learning outcomes [31] Nevertheless, ESNs still acknowledged the benefits of the SBE program, suggesting that balancing psychological safety with rigorous training can yield positive outcomes [32,33]

The negative impact of *COVID-19* on the SBE program has not only been experienced at this centre but across simulation centres worldwide [32] Everywhere, nursing education faced unprecedented challenges in maintaining the quality of both clinical and SBE due to the pandemic [33]

## Limitations

Initially, the course was set to follow the structure originally designed for VSTs, enforcing strict passing standards without concurrent feedback but with assessments at the end of every simulation session. Only a few ESNs were exposed to the initial concept, which did not impact the program results. Due to resource limitations, a single instructor performed both pre- and post-course assessment, introducing a risk of bias. This study only recruited a limited number of participants from a single department, which may restrict generalisability [34] Focus group interviews risk consensus bias through group consensus, but offer significant information power for new insights. Surgeons working with the ESNs were not interviewed, which could have provided an additional perspective and further contextualised the nurses’ experiences. While this study has clear limitations, its strengths are notable. The potential for implementing role-reversal training on a larger scale is substantial, as many doctor/surgeon-specific SBE programs already exist and can be easily amended to target nurses.

## Conclusion

The role-reversal simulation-based education program for ESNs improved technical skills and strengthened teamwork and collaboration in the operating room. Participants reported a greater understanding of procedural workflows and an increased ability to anticipate surgeons’ needs, which fostered a more cohesive and efficient team dynamic. These findings highlight the potential of role-reversal training to enhance surgical performance and job satisfaction among OR staff and support its integration into future interdisciplinary training initiatives.

## AI declaration

During the preparation of this manuscript, the authors used ChatGPT (OpenAI) exclusively for language refinement (grammar, style, clarity). No AI tools were used to generate scientific content, analyze or interpret data, or draw conclusions. All content, intellectual work, and responsibility for accuracy and integrity remain with the human authors.

## Data sharing statement

In accordance with Danish legislation and the European General Data Protection Regulation (GDPR), the datasets generated and analysed during the current study (including quantitative and qualitative data) contain potentially identifiable information and cannot be made publicly available.

Data may, however, be shared under a formal data processing agreement between the data controller and the requesting institution. Such agreements can be arranged by contacting the corresponding author.

## Funding

This research did not receive any specific grant from funding agencies in the public, commercial, or not-for-profit sectors. The simulator was provided by Angiomentor (Surgical Science, Sweden). IVH additionally reports support from a Senior Clinical Fellowship (802,314 N) of the Research Foundation – Flanders (FWO).

## CRediT authorship contribution statement

**Jonathan Lawaetz:** Writing – review & editing, Writing – original draft, Visualization, Validation, Supervision, Software, Project administration, Methodology, Investigation, Data curation, Conceptualization. **Maj Siercke:** Writing – review & editing, Methodology, Investigation, Data curation, Conceptualization. **Rebecca Skov:** Writing – review & editing, Methodology, Investigation, Data curation. **Lars Konge:** Writing – review & editing, Visualization, Validation, Resources, Funding acquisition, Conceptualization. **Isabelle Van Herzeele:** Writing – review & editing, Validation, Supervision, Methodology, Data curation, Conceptualization. **Jonas Peter Eiberg:** Writing – review & editing, Visualization, Supervision, Resources, Methodology, Conceptualization.

## Declaration of competing interest

The authors declare the following financial interests/personal relationships which may be considered as potential competing interests:

Jonathan Lawaetz reports equipment, drugs, or supplies was provided by Angiomentor, Surgical Science. Isabelle Van Herzeele reports a relationship with Research Foundation, Flanders, Belgium that includes: funding grants. The other authors declare that they have no known competing financial interests or personal relationships that could have appeared to influence the work reported in this paper.

## Appendix Supplementary materials

Image, application 1

Image, application 2
