# Supplementary material for: A simulation-based role-reversal training program improves knowledge, skills, and job satisfaction of endovascular scrub nurses
Source: Surg Pract Sci. 2026 Feb 19;25:100335. doi: 10.1016/j.sipas.2026.100335 (PMC12969722; doi:10.1016/j.sipas.2026.100335)
Supplement: Supplementary file 2 [file mmc2.docx]

STROBE Statement—checklist of items that should be included in reports of observational studies

|  | Item No. | Recommendation | Page  No. | Relevant text from manuscript |
| --- | --- | --- | --- | --- |
| **Title and abstract** | 1 | (*a*) Indicate the study’s design with a commonly used term in the title or the abstract | 1 | Title: “A simulation-based role-reversal training program improves knowledge, skills, and job satisfaction of endovascular scrub nurses.” Abstract: states “prospective single-centre mixed-methods study” and summarises quantitative and qualitative results. |
|  |  | (*b*) Provide in the abstract an informative and balanced summary of what was done and what was found | 1 | This study assessed the effectiveness of a modified simulation-based education (SBE) program, originally designed for vascular surgical trainees, adapted for ESNs.”“Qualitative findings, derived from the themes of learning potential, job satisfaction, culture, feedback, overwhelming, and COVID-19, demonstrated improved understanding of procedural sequences, teamwork, and job satisfaction. |
| Introduction | | | |  |
| Background/rationale | 2 | Explain the scientific background and rationale for the investigation being reported | 3 | Growing demands for patient safety and outcomes require highly skilled surgical teams, where scrub nurses play a central role in ensuring efficiency and safety. Well-tuned teamwork in the OR is essential for patient safety and rational use of resources; the growing complexity of surgical procedures and increased implementation of new technologies further compound this… Simulation-based education (SBE) effectively improves endovascular competence among vascular trainees and is increasingly integrated into training curricula. SBE combines procedural training and teamwork in a safe environment, improving both technical and interpersonal skills. |
| Objectives | 3 | State specific objectives, including any prespecified hypotheses |  | This study aims to assess the effectiveness of an adapted interprofessional endovascular skills training program for experienced ESNs in terms of improvement in technical and non-technical abilities, and it seeks to understand the derivative effects of such a program on daily activities and teamwork in the actual OR. |
| Methods | | | |  |
| Study design | 4 | Present key elements of study design early in the paper | 4 | This prospective single-centre mixed-methods study evaluated a simulation-based role-reversal training program for endovascular scrub nurses (ESNs) between March 2020 and March 2021. |
| Setting | 5 | Describe the setting, locations, and relevant dates, including periods of recruitment, exposure, follow-up, and data collection | 4 | “This prospective single-centre mixed-methods study evaluated a simulation-based role-reversal training program for endovascular scrub nurses (ESNs) between March 2020 and March 2021.”  “The study included experienced ESNs, all employed at the same Department of Vascular Surgery with approximately 450 endovascular procedures for lower-limb ischemia annually.  The SBE program in basic endovascular skills was initially designed for VSTs at the onset of their endovascular career. The program is derived from … (PRO SPECT) and consists of four modules incorporating e-learning and simulation cases. Three hours were allocated to each hands-on simulation module.” |
| Participants | 6 | (*a*) *Cohort study*—Give the eligibility criteria, and the sources and methods of selection of participants. Describe methods of follow-up  *Case-control study*—Give the eligibility criteria, and the sources and methods of case ascertainment and control selection. Give the rationale for the choice of cases and controls  *Cross-sectional study*—Give the eligibility criteria, and the sources and methods of selection of participants | 4 | The study included experienced ESNs, all employed at the same Department of Vascular Surgery with approximately 450 endovascular procedures for lower-limb ischemia annually. Each ESN had assisted approximately 100 PAD procedures within the last 2–3 years. Only experienced ESNs were included to ensure a uniform baseline of procedural familiarity and to allow the training to focus on role-reversal understanding and interdisciplinary teamwork rather than basic scrub-nurse competencies. |
|  |  | (*b*) *Cohort study*—For matched studies, give matching criteria and number of exposed and unexposed  *Case-control study*—For matched studies, give matching criteria and the number of controls per case |  |  |
| Variables | 7 | Clearly define all outcomes, exposures, predictors, potential confounders, and effect modifiers. Give diagnostic criteria, if applicable | 5 | Knowledge assessment was done with standardised multiple-choice questionnaires (MCQ) pre- and post-course and at the end of each module. As in the original PROSPECT programme, passing each module’s MCQ was required before progression. In contrast, benchmark performance in simulation cases was not mandatory. Technical skills were evaluated pre- and post-course with a standardised simulation case on a common iliac artery lesion. They were assessed by the same instructor using a Global Rating Scale (GRS) and an Examiner’s Checklist, both rated 1–5 with a maximum of 55 and 85 points, respectively. |
| Data sources/ measurement | 8* | For each variable of interest, give sources of data and details of methods of assessment (measurement). Describe comparability of assessment methods if there is more than one group | 5 | Knowledge assessment was done with standardised multiple-choice questionnaires (MCQ) pre- and post-course and at the end of each module. As in the original PROSPECT programme, passing each module’s MCQ was required before progression.  Technical skills were evaluated pre- and post-course with a standardised simulation case on a common iliac artery lesion. They were assessed by the same instructor using a Global Rating Scale (GRS) and an Examiner’s Checklist, both rated 1–5 with a maximum of 55 and 85 points, respectively. |
| Bias | 9 | Describe any efforts to address potential sources of bias | 4 | Only experienced ESNs were included to ensure a uniform baseline of procedural familiarity and to allow the training to focus on role-reversal understanding and interdisciplinary teamwork rather than basic scrub-nurse competencies. |
| Study size | 10 | Explain how the study size was arrived at | 4 | The study included experienced ESNs, all employed at the same Department of Vascular Surgery with approximately 450 endovascular procedures for lower-limb ischemia annually  **Clarification** (not in manuscript): All experienced ESNs from the department were invited, and all available staff participated (n = 11). No formal sample size calculation was performed, as the study population represented the entire accessible group of eligible participants. |

Continued on next page

| Quantitative variables | 11 | Explain how quantitative variables were handled in the analyses. If applicable, describe which groupings were chosen and why | 5-6 | All analyses were performed in R (version 4.4.0; R Foundation for Statistical Computing, Vienna, Austria) using RStudio (version 2024.12.0+467; Posit Software, PBC). As the Global Rating Scale (GRS) and Examiner’s Checklist are ordinal and the sample size was limited, normality was not assumed, and data were summarised as median [IQR]. Pre- and post-course comparisons were conducted using the Wilcoxon signed-rank test for paired data. All tests were two-sided, and p < 0.05 was considered statistically significant. |
| --- | --- | --- | --- | --- |
| Statistical methods | 12 | (*a*) Describe all statistical methods, including those used to control for confounding | 5-6 | All analyses were performed in R (version 4.4.0; R Foundation for Statistical Computing, Vienna, Austria) using RStudio (version 2024.12.0+467; Posit Software, PBC). As the Global Rating Scale (GRS) and Examiner’s Checklist are ordinal and the sample size was limited, normality was not assumed, and data were summarised as median [IQR]. Pre- and post-course comparisons were conducted using the Wilcoxon signed-rank test for paired data.  **Clarification** (not in manuscript):  No confounders were included, as all participants received the same intervention and acted as their own controls in paired pre/post analyses. |
|  |  | (*b*) Describe any methods used to examine subgroups and interactions |  | (Not applicable — no subgroup analyses described)  **Clarification** (not in manuscript):  No subgroup or interaction analyses were conducted due to the small and homogenous participant group. |
|  |  | (*c*) Explain how missing data were addressed |  | (Not explicitly stated)  **Clarification** (not in manuscript):  No missing data were reported for pre/post assessment |
|  |  | (*d*) *Cohort study*—If applicable, explain how loss to follow-up was addressed  *Case-control study*—If applicable, explain how matching of cases and controls was addressed  *Cross-sectional study*—If applicable, describe analytical methods taking account of sampling strategy |  | (Not applicable — single-session intervention) |
|  |  | (*e*) Describe any sensitivity analyses |  | (Not performed or reported)  **Clarification** (not in manuscript):  No sensitivity analyses were conducted, as the study aimed to provide descriptive paired comparisons within a small cohort. |
| Results | | | | |
| Participants | 13* | (a) Report numbers of individuals at each stage of study—eg numbers potentially eligible, examined for eligibility, confirmed eligible, included in the study, completing follow-up, and analysed | 6 | This study included 11 ESNs. The results are divided into quantitative and qualitative data. |
|  |  | (b) Give reasons for non-participation at each stage |  | Not applicable |
|  |  | (c) Consider use of a flow diagram |  | Not applicable |
| Descriptive data | 14* | (a) Give characteristics of study participants (eg demographic, clinical, social) and information on exposures and potential confounders |  | **Clarification** (not in manuscript):  Participant demographic and clinical characteristics were homogeneous; all 11 were experienced endovascular scrub nurses employed at the same vascular surgery department, each with comparable procedural exposure (~100 PAD cases within 2–3 years). Because participants were nearly identical in role and experience, no additional descriptive variables were reported in the Results section. |
|  |  | (b) Indicate number of participants with missing data for each variable of interest |  | No missing data |
|  |  | (c) *Cohort study*—Summarise follow-up time (eg, average and total amount) |  | **Clarification** (not in manuscript):  The study did not include longitudinal follow-up. Pre- and post-intervention data were collected within the same short intervention period (1–2 months). |
| Outcome data | 15* | *Cohort study*—Report numbers of outcome events or summary measures over time | 6 | The pre- and post-MCQ scores significantly increased from median [IQR] 12.0 [11.0; 13.0] to 14.0 [13.0; 16.0] (p = .012).  Significant improvement was also observed in technical skills, as evidenced by the Global Rating Scale (GRS) with an increase from median [IQR] 19.0 [14.0; 24.0] to 43.0 [32.0; 44.0] (p = .004), the Examiner’s Checklist with an increase from median [IQR] 49.0 [45.0; 58.0] to 73.0 [65.0; 78.0] (p = .004), and reduction in the Procedure and Fluoroscopy times from median [IQR] 39.2 [28.0; 42.5] to 18.4 [16.6; 21.5] minutes and 21.0 [14.2; 27.5] to 10.1 [9.1; 13.2] minutes, respectively (p = .004 and p = .005) |
|  |  | *Case-control study—*Report numbers in each exposure category, or summary measures of exposure |  |  |
|  |  | *Cross-sectional study—*Report numbers of outcome events or summary measures |  |  |
| Main results | 16 | (*a*) Give unadjusted estimates and, if applicable, confounder-adjusted estimates and their precision (eg, 95% confidence interval). Make clear which confounders were adjusted for and why they were included |  | The pre- and post-MCQ scores significantly increased from median [IQR] 12.0 [11.0; 13.0] to 14.0 [13.0; 16.0] (p = .012).  Significant improvement was also observed in technical skills, as evidenced by the Global Rating Scale (GRS) with an increase from median [IQR] 19.0 [14.0; 24.0] to 43.0 [32.0; 44.0] (p = .004), the Examiner’s Checklist with an increase from median [IQR] 49.0 [45.0; 58.0] to 73.0 [65.0; 78.0] (p = .004), and reduction in the Procedure and Fluoroscopy times from median [IQR] 39.2 [28.0; 42.5] to 18.4 [16.6; 21.5] minutes and 21.0 [14.2; 27.5] to 10.1 [9.1; 13.2] minutes, respectively (p = .004 and p = .005) |
|  |  | (*b*) Report category boundaries when continuous variables were categorized |  | Not applicable |
|  |  | (*c*) If relevant, consider translating estimates of relative risk into absolute risk for a meaningful time period |  | Not applicable |

Continued on next page

| Other analyses | 17 | Report other analyses done—eg analyses of subgroups and interactions, and sensitivity analyses |  | Not applicable |
| --- | --- | --- | --- | --- |
| Discussion | | | | |
| Key results | 18 | Summarise key results with reference to study objectives | 9 | The ESNs demonstrated significant improvement in knowledge and technical skills following the SBE. This was evident in key areas such as procedure and fluoroscopy times, GRS, Examiner’s Checklist and MCQs. Two novel aspects of this study were the clinical applicability of the principle of role reversal training and the fact that the ESNs’ improvement was comparable to that of VSTs, the SBE program’s original target. |
| Limitations | 19 | Discuss limitations of the study, taking into account sources of potential bias or imprecision. Discuss both direction and magnitude of any potential bias | 12 | Initially, the course was set to follow the structure originally designed for VSTs, enforcing strict passing standards without concurrent feedback but with assessments at the end of every simulation session. Only a few ESNs were exposed to the initial concept, which did not impact the program results. Due to resource limitations, a single instructor performed both pre- and post-course assessment, introducing a risk of bias. This study only recruited a limited number of participants from a single department, which may restrict generalisability. Focus group interviews risk consensus bias through group consensus, but offer significant information power for new insights. Surgeons working with the ESNs were not interviewed, which could have provided an additional perspective and further contextualised the nurses’ experiences. |
| Interpretation | 20 | Give a cautious overall interpretation of results considering objectives, limitations, multiplicity of analyses, results from similar studies, and other relevant evidence | 10-11 | “Beyond the improvements in knowledge and technical skills, the ESNs also reported better non-technical abilities, such as anticipation, communication, and teamwork… Similar effects have been observed by Gowda et al.… and by Satkunanatham and Sechachalam… This phenomenon—technical training improving non-technical performance—has been further supported by Brunckhorst et al. and Hull et al., highlighting a strong correlation between technical proficiency and teamwork.”  “Adapting the training to include concurrent feedback and altering the passing requirements proved beneficial for the overall reception of the program… This suggests that feedback methods may need to be tailored to the training context and the learners’ backgrounds.” |
| Generalisability | 21 | Discuss the generalisability (external validity) of the study results | 11 | This study only recruited a limited number of participants from a single department, which may restrict generalisability. While this study has clear limitations, its strengths are notable. The potential for implementing role-reversal training on a larger scale is substantial, as many doctor/surgeon-specific SBE programs already exist and can be easily amended to target nurses. |
| Other information | |  | | |
| Funding | 22 | Give the source of funding and the role of the funders for the present study and, if applicable, for the original study on which the present article is based | 6 | This research did not receive any specific grant from funding agencies in the public, commercial, or not-for-profit sectors. The simulator was provided by Angiomentor (Surgical Science, Sweden). |

*Give information separately for cases and controls in case-control studies and, if applicable, for exposed and unexposed groups in cohort and cross-sectional studies.

**Note:** An Explanation and Elaboration article discusses each checklist item and gives methodological background and published examples of transparent reporting. The STROBE checklist is best used in conjunction with this article (freely available on the Web sites of PLoS Medicine at http://www.plosmedicine.org/, Annals of Internal Medicine at http://www.annals.org/, and Epidemiology at http://www.epidem.com/). Information on the STROBE Initiative is available at www.strobe-statement.org.
